# Knowledge and Determinants of Behavioral Responses to the Pandemic of COVID-19

**DOI:** 10.3389/fmed.2021.673187

**Published:** 2021-06-09

**Authors:** Gang Lv, Jing Yuan, Stephanie Hsieh, Rongjie Shao, Minghui Li

**Affiliations:** ^1^Department of General Surgery, The 1st Medical Center of Chinese PLA General Hospital, Beijing, China; ^2^Department of Clinical Pharmacy and Pharmacy Administration, School of Pharmacy, Fudan University, Shanghai, China; ^3^Department of Pharmacy, Scarborough Health Network – Centenary Hospital, Scarborough, ON, Canada; ^4^Department of Health Economics, China Pharmaceutical University, Nanjing, China; ^5^Department of Clinical Pharmacy and Translational Science, University of Tennessee Health Science Center, Memphis, TN, United States

**Keywords:** COVID-19, knowledge, determinants of health, preventive health behaviors, pharmacist, student

## Abstract

**Background:** Understanding knowledge and behavioral responses to the pandemic of coronavirus disease 2019 (COVID-19) is important for appropriate public health interventions.

**Objectives:** To assess knowledge of COVID-19 and to examine determinants associated with the adoption of preventive health behaviors among future health care providers.

**Methods:** An anonymous online survey was sent out to pharmacy students in high and low-endemic areas of COVID-19 in China. Based on recommendations from the Chinese Center for Disease Control and Prevention, preventive health behaviors examined in this study included washing hands, wearing a face mask, and maintaining social distancing. The Health Belief Model (HBM) was used and measured by a seven-point Likert scale (one as extremely unlikely; seven as extremely likely). Multivariate linear regression models were used to examine predictors of preventive health behaviors.

**Results:** Among 203 respondents who finished the survey, a medium level of knowledge (4.41 ± 0.95) of COVID-19 was reported. Respondents were extremely likely to wear a face mask (6.85 ± 0.60), but only moderately likely to engage in washing hands (5.95 ± 1.38) and maintaining social distancing (6.19 ± 1.60). Determinants of washing hands were cue to action, self-efficacy, knowledge, and gender; wearing a face mask were cue to action, self-efficacy, knowledge, and ethnicity; and maintaining social distancing were cue to action and self-efficacy.

**Conclusions:** Public health interventions should consider incorporating cue to action, self-efficacy, and knowledge as factors to potentially improve the adoption of face mask-wearing, hand washing, and social distancing as appropriate individual preventive measures, especially if local and regional authorities are considering reopening schools sometime in future.

## Introduction

The coronavirus disease 2019 (COVID-19) is a new, rapidly spreading infectious disease caused by the “SARS-CoV-2” virus. World Health Organization (WHO) has declared COVID-19 as a pandemic (1). The first case of COVID-19 was reported in Wuhan, China in December 2019 (1). Since then, it has spread globally at an alarming rate. National and regional authorities have implemented different prevention and control measures to contain the quick spread of COVID-19. Individuals also have different behavioral responses to the pandemic of COVID-19.

Government responses to COVID-19 have included implementing lockdowns, canceling public gatherings, imposing travel restrictions, and closing schools. The effectiveness of these measures has been debated in the literature. The implementation of lockdown and travel restrictions was found to be effective in reducing new infections (2, 3). School closures might lead to a reduction in mortality (4). Restricting travels from areas with active outbreaks could reduce disease transmission if combined with public health interventions and individual behavior changes (5).

To encourage individual behavior changes during the pandemic of COVID-19, both local and global health organizations have released recommendations on individual preventive measures to reduce disease transmissibility. World Health Organization recommends frequent hand washing, social distancing, avoidance of face touching, practicing respiratory hygiene, staying home, and seeking medical care early if there are suspected symptoms (6). The Centers for Disease Control and Prevention (CDC) in different countries have similar recommendations on hand washing and social distancing, but have different opinions on face mask-wearing (7, 8). However, little is known about whether individuals have changed their behaviors based on recommendations from health organizations.

This study used the Health Belief Model (HBM) as the conceptual framework. The HBM is one of the most widely used psychosocial models to examine factors associated with individual behavior (9). Key components of the HBM included perceived susceptibility (the risk of getting a disease), perceived severity (the seriousness of a disease), perceived benefits (the gains to perform a behavior), perceived barriers (the obstacles to perform a behavior), and cue to action (the stimulus to perform a behavior) (9). Recent modifications of the HBM have added other components of self-efficacy (the level of confidence to perform a behavior) and knowledge (the level of expertise in an area) (10, 11).

The HBM has been used to assess predictors of adopting preventive health behaviors in previous infectious disease outbreaks. Perceived susceptibility significantly influenced the adoption of preventive health behaviors during the severe acute respiratory syndrome (SARS) and H1N1 outbreaks (12–15). Perceived severity significantly influenced the adoption of preventive health behaviors during the H1N1 outbreak (16, 17). Perceived benefits significantly influenced the adoption of preventive health behaviors during the SARS and H1N1 outbreaks (12, 14–17). Perceived barriers significantly influenced the adoption of preventive health behaviors during the H1N1 outbreak (15–17). Cues to action significantly influenced the adoption of preventive health behaviors during the SARS, H1N1, and Zika virus outbreaks (12–15, 18). Self-efficacy was not associated with the adoption of preventive health behaviors during the SARS and H1N1 outbreaks (13, 15). Knowledge did not influence the adoption of preventive health behaviors during the H1N1 and SARS outbreak (13, 16).

Understanding individuals' knowledge and behavioral responses to the pandemic of COVID-19 is important to contain the outbreak. However, there is a paucity of studies available on the knowledge and preventive health behaviors of COVID-19. Therefore, to address these literature gaps, this study aimed to (1) to assess individuals' knowledge of COVID-19 and (2) to examine determinants associated with the potential adoption of preventive health behaviors, including washing hands, wearing a face mask, and maintaining social distancing, among future pharmacists.

## Methods

### Study Participants

This study was conducted in March 2020 in China. During that time, China had slowed the spread of COVID-19 and saw continuous decreases in the newly confirmed cases. In low-endemic areas, most prevention and control measures implemented by local authorities were discontinued (e.g., lockdowns and travel restrictions). However, school closures were still in effect in most parts of China. Specifically, all colleges and universities were still closed and classes were offered online. A significant challenge for schools to reopen is that students might not have enough knowledge and motivation to engage in preventive health behaviors to prevent contracting and spreading COVID-19 in the community.

A pilot study was conducted on eight respondents to test the clarity of survey questions and confusing questions were revised. An anonymous online survey was sent to pharmacy students who lived in high and low-endemic areas in China. We sent surveys to 800 pharmacy students who were 18 years and older from four different colleges/universities, including both comprehensive and professional colleges/universities. High-endemic areas included Hubei, Guangdong, Henan, Zhejiang, and Hunan, where the total number of COVID-19 cases were 1,000 and higher; low-endemic areas included provinces with the total number of COVID-19 cases lower than 1,000. This study was approved by the University institutional review board.

### Measurement

The survey collected information on knowledge of COVID-19, preventive health behaviors, and demographics. Respondents' knowledge was assessed by when and how they first heard of COVID-19. This study also tested if respondents knew how COVID-19 was transmitted and of the availability of test kit, medication, and vaccine for COVID-19. Demographics collected in the survey included age, gender, ethnicity, and residence area (high vs. low-endemic areas).

Preventive health behaviors examined in this study included washing hands, wearing a face mask, and maintaining social distancing as recommended by the China CDC (8). Respondents were asked to indicate their likelihood of performing these preventive health behaviors and measured by a seven-point Likert scale (one as extremely unlikely; seven as extremely likely). Components of the HBM were also measured by a seven-point Likert scale (one as strongly disagree; seven as strongly agree). Perceived susceptibility was assessed by whether respondents believe that they are at risk of getting COVID-19. Perceived severity was assessed by their thoughts of COVID-19 as a serious illness. Perceived benefits were assessed by whether respondents believe each preventive health behavior can prevent COVID-19. Perceived barriers were assessed by whether they believe it is inconvenient to perform each preventive health behavior. Cue to action was assessed by whether respondents would perform each preventive health behavior if recommended. Self-efficacy was assessed by whether they are able to perform each preventive health behavior. Knowledge was assessed by whether respondents have enough knowledge about COVID-19 and defined as low (score 1–2), medium (score 3–5), and high (score 6–7) levels.

### Data Analysis

We excluded study participants in the data analysis if they reported extreme answers, defined as having the same response to all survey questions. Preventive health behaviors and components of the HBM were analyzed as means and compared between different demographic groups using independent sample *t*-tests. Pearson correlation coefficients were calculated to examine associations between different preventive health behaviors. To predict preventive health behaviors, three multivariate linear regression models were used. In each model, the dependent variable was the preventive health behavior and independent variables included components of the HBM and demographic variables. All statistical analyses were conducted using SAS version 9.4 (SAS Institute, Cary, NC).

## Results

Among 203 respondents who finished the survey (response rate of 25.38%), the majority of them were aged 18–22 years (54.68%), female (69.95%), of Han ethnicity (91.13%), and lived in low-endemic areas (70.44%) (Table 1). For the knowledge of COVID-19, most of respondents first heard of COVID-19 in January 2020 (59.61%). The two most common sources of this information were websites (84.73%) and social media (59.11%). Almost all respondents knew that COVID-19 was transmitted through droplets (98.52%) and two-thirds of them also knew that close contact (66.50%) could transmit the virus. For the availability of different medical products, the majority of respondents thought that we have test kits to detect (79.31%) but do not have a vaccine to prevent COVID-19 (78.82%). Approximately half of respondents thought that there is no medication to treat COVID-19 (52.22%).

**Table 1:** Characteristics and knowledge of study respondents (*N* = 203).

	***N***	**%**
**Demographics**		
**Age**		
18–22	111	54.68
23+	92	45.32
**Gender**		
Female	142	69.95
Male	61	30.05
**Ethnicity**		
Han	185	91.13
Minority	18	8.87
**Area**		
Low-endemic area	143	70.44
High-endemic area	60	29.56
**First Time Heard of COVID-19**		
2019	75	36.95
January 2020	121	59.61
February 2020 and later	7	3.45
**Source of Information**		
TV	49	24.14
Website	172	84.73
Social media	120	59.11
Radio	9	4.43
In print	7	3.45
Family/friend	54	26.60
**Method of Transmission**		
Close contact	135	66.50
Droplets	200	98.52
Bodily fluids	90	44.33
Sex	79	38.92
**Availability of Test Kit**		
Yes	161	79.31
No	23	11.33
Unsure	19	9.36
**Availability of Medication**		
Yes	56	27.59
No	106	52.22
Unsure	41	20.20
**Availability of Vaccine**		
Yes	15	7.39
No	160	78.82
Unsure	28	13.79

For preventive health behaviors, respondents reported being extremely likely to wear a face mask (6.85 ± 0.60), but only moderately likely to engage in hand washing (5.95 ± 1.38) and social distancing (6.19 ± 1.60) (Table 2). For Pearson correlation coefficients, three preventive health behaviors were positively correlated with each other (washing hands vs. wearing a face mask: *r* = 0.24, *p* < 0.01; washing hands vs. maintaining social distancing: *r* = 0.24, *p* < 0.01; wearing a face mask vs. maintaining social distancing: *r* = 0.31, *p* < 0.01). Different demographic groups had a similar likelihood of performing preventive health behaviors.

**Table 2:** Preventive health behaviors of study respondents by age, gender, ethnicity, and residence area.

	**Overall**	**Age**			**Gender**			**Ethnicity**			**Residence area**		
		**18–22**	**23+**	***P***	**Female**	**Male**	***P***	**Han**	**Minority**	***P***	**Low-endemic**	**High-endemic**	***P***
	**Mean ± SD^†^**	**Mean ± SD**	**Mean ± SD**		**Mean ± SD**	**Mean ± SD**		**Mean ± SD**	**Mean ± SD**		**Mean ± SD**	**Mean ± SD**	
**Preventive health behavior of**													
Washing hands	5.95 ± 1.38	6.02 ± 1.29	5.87 ± 1.48		6.02 ± 1.34	5.79 ± 1.45		5.91 ± 1.40	6.33 ± 1.03		5.94 ± 1.37	5.97 ± 1.41	
Wearing a face mask	6.85 ± 0.60	6.88 ± 0.51	6.82 ± 0.69		6.82 ± 0.64	6.90 ± 0.47		6.86 ± 0.57	6.72 ± 0.83		6.85 ± 0.54	6.83 ± 0.71	
Maintaining social distancing	6.19 ± 1.60	6.39 ± 1.36	5.96 ± 1.84	^*^	6.23 ± 1.57	6.11 ± 1.68		6.23 ± 1.56	5.83 ± 2.04		6.14 ± 1.63	6.32 ± 1.56	
**Perceived susceptibility of COVID-19**	2.92 ± 1.70	3.05 ± 1.72	2.75 ± 1.67		3.08 ± 1.65	2.52 ± 1.77	^**^	2.91 ± 1.70	3.00 ± 1.78		2.88 ± 1.68	3.00 ± 1.76	
**Perceived severity of COVID-19**	5.08 ± 1.51	5.00 ± 1.50	5.17 ± 1.53		4.96 ± 1.46	5.36 ± 1.60	^*^	5.11 ± 1.53	4.72 ± 1.27		5.17 ± 1.46	4.85 ± 1.61	
**Perceived benefits of**													
Washing hands	5.50 ± 1.27	5.47 ± 1.23	5.54 ± 1.32		5.44 ± 1.29	5.64 ± 1.24		5.50 ± 1.22	5.50 ± 1.76		5.51 ± 1.19	5.48 ± 1.47	
Wearing a face mask	5.87 ± 1.23	5.97 ± 1.15	5.74 ± 1.32		5.83 ± 1.24	5.95 ± 1.22		5.82 ± 1.26	6.33 ± 0.77	^**^	5.86 ± 1.19	5.88 ± 1.33	
Maintaining social distancing	6.19 ± 0.93	6.25 ± 0.86	6.11 ± 1.01		6.18 ± 0.95	6.20 ± 0.89		6.18 ± 0.91	6.22 ± 1.12		6.18 ± 0.95	6.20 ± 0.88	
**Perceived barriers to**													
Washing hands	2.65 ± 1.69	2.47 ± 1.63	2.87 ± 1.76	^*^	2.68 ± 1.63	2.59 ± 1.86		2.73 ± 1.72	1.78 ± 1.06	^***^	2.62 ± 1.71	2.72 ± 1.67	
Wearing a face mask	5.17 ± 1.66	5.17 ± 1.68	5.17 ± 1.64		5.23 ± 1.57	5.03 ± 1.86		5.26 ± 1.60	4.22 ± 2.02	^**^	5.13 ± 1.70	5.28 ± 1.56	
Maintaining social distancing	2.58 ± 1.76	2.33 ± 1.68	2.87 ± 1.83	^**^	2.63 ± 1.75	2.46 ± 1.79		2.59 ± 1.75	2.39 ± 1.88		2.57 ± 1.76	2.60 ± 1.78	
**Cue to action of**													
Washing hands	6.27 ± 1.03	6.38 ± 1.01	6.13 ± 1.04	^*^	6.27 ± 1.03	6.26 ± 1.03		6.23 ± 1.06	6.61 ± 0.61	^**^	6.24 ± 0.99	6.32 ± 1.13	
Wearing a face mask	6.59 ± 0.72	6.69 ± 0.55	6.47 ± 0.87	^**^	6.57 ± 0.69	6.64 ± 0.80		6.57 ± 0.73	6.78 ± 0.55		6.56 ± 0.75	6.67 ± 0.65	
Maintaining social distancing	6.28 ± 1.18	6.29 ± 1.27	6.26 ± 1.07		6.17 ± 1.29	6.52 ± 0.83	^**^	6.28 ± 1.15	6.22 ± 1.52		6.28 ± 1.13	6.27 ± 1.29	
**Self-efficacy of**													
Washing hands	5.84 ± 1.30	5.95 ± 1.26	5.71 ± 1.34		5.84 ± 1.33	5.84 ± 1.24		5.79 ± 1.32	6.33 ± 0.91	^*^	5.80 ± 1.23	5.93 ± 1.50	
Wearing a face mask	6.60 ± 0.70	6.70 ± 0.53	6.47 ± 0.84	^**^	6.59 ± 0.73	6.61 ± 0.64		6.58 ± 0.72	6.78 ± 0.43	^*^	6.61 ± 0.65	6.57 ± 0.81	
Maintaining social distancing	6.07 ± 1.13	6.17 ± 1.04	5.95 ± 1.23		6.08 ± 1.16	6.03 ± 1.06		6.05 ± 1.11	6.28 ± 1.32		6.06 ± 1.15	6.08 ± 1.09	
**Knowledge of COVID-19**	4.41 ± 0.95	4.32 ± 0.94	4.52 ± 0.95		4.30 ± 0.86	4.69 ± 1.09	^**^	4.38 ± 0.94	4.72 ± 0.96		4.44 ± 0.98	4.35 ± 0.88	

† *1 as extremely unlikely and 7 as extremely likely.*

* *Significant at 0.1,*

** *Significant at 0.05,*

*** *Significant at 0.01*.

For components of the HBM, respondents reported a slightly low perceived susceptibility (2.92 ± 1.70) of COVID-19 and males reported a lower level of susceptibility (Table 2). Respondents reported a slightly high perceived severity (5.08 ± 1.51) of COVID-19. Perceived benefits were moderately high for all preventive health behaviors (washing hands: 5.50 ± 1.27; wearing a face mask: 5.87 ± 1.23; and maintaining social distancing: 6.19 ± 0.93) and ethnic minorities reported a higher level of benefit of wearing a face mask. Perceived barriers were slightly low to wash hands (2.65 ± 1.69) and maintain social distancing (2.58 ± 1.76). Ethnic minorities reported a lower level of barrier to wash hands and those aged 23 years and older reported a higher level of barrier to maintain social distancing. Perceived barriers to wear a face mask were slightly high (5.17 ± 1.66) and ethnic minorities reported a lower level of barrier. Cue to action was moderately high for washing hands (6.27 ± 1.03) and maintaining social distancing (6.28 ± 1.18). Ethnic minorities reported a higher level of cue to action of washing hands and males reported a higher level of cue to action of maintaining social distancing. Cue to action of wearing a face mask was extremely high (6.59 ± 0.72) and those aged 23 years and older reported a lower level of cue to action. Self-efficacy was moderately high for washing hands (5.84 ± 1.30) and maintaining social distancing (6.07 ± 1.13). Self-efficacy of wearing a face mask was extremely high (6.60 ± 0.70) and those aged 23 years and older reported a lower level of self-efficacy. Respondents reported a medium level of knowledge (4.41 ± 0.95) of COVID-19 and males reported a higher level of knowledge.

In the multivariate regression models, determinants of washing hands included cue to action (β: 0.27; 95% CI: 0.05 to 0.48), self-efficacy (β: 0.56; 95% CI: 0.39 to 0.73), and knowledge (β: 0.15; 95% CI: 0.01 to 0.31) and being a male was negatively associated with washing hands (β: −0.35; 95% CI: −0.68 to −0.03) (Table 3). Determinants of wearing a face mask included cue to action (β: 0.20; 95% CI: 0.06 to 0.34), self-efficacy (β: 0.21; 95% CI: 0.06 to 0.35), and knowledge (β: 0.09; 95% CI: 0.01 to 0.18) and being an ethnic minority was negatively associated with wearing a face mask (β: −0.32; 95% CI: −0.60 to −0.03). Determinants of maintaining social distancing included cue to action (β: 0.19; 95% CI: 0.01 to 0.38) and self-efficacy (β: 0.43; 95% CI: 0.19 to 0.66).

**Table 3:** Multivariate linear regressions on preventive health behaviors.

	**Washing hands**			**Wearing a face mask**			**Maintaining social distancing**		
	**β**	**95% CI**		**β**	**95% CI**		**β**	**95% CI**	
**Health Belief Model**									
Perceived susceptibility	0.03	−0.06	0.11	0.00	−0.05	0.04	0.08	−0.05	0.21
Perceived severity	0.01	−0.08	0.11	0.00	−0.05	0.05	0.04	−0.10	0.18
Perceived benefits	0.02	−0.10	0.13	0.04	−0.02	0.10	0.10	−0.15	0.34
Perceived barriers	−0.01	−0.10	0.07	−0.01	−0.06	0.03	−0.02	−0.15	0.12
Cue to action	0.27	0.05	0.48	0.20	0.06	0.34	0.19	0.01	0.38
Self-efficacy	0.56	0.39	0.73	0.21	0.06	0.35	0.43	0.19	0.66
Knowledge	0.15	0.01	0.31	0.09	0.01	0.18	0.01	−0.22	0.23
**Demographics**									
**Age**									
18–22	Ref			Ref			Ref		
23+	0.09	−0.20	0.38	0.00	−0.16	0.16	−0.33	−0.76	0.11
**Gender**									
Female	Ref			Ref			Ref		
Male	−0.35	−0.68	−0.03	−0.04	−0.21	0.14	−0.12	−0.61	0.38
**Ethnicity**									
Han	Ref			Ref			Ref		
Minority	−0.18	−0.71	0.34	−0.32	−0.60	−0.03	−0.60	−1.37	0.18
**Residence area**									
Low-endemic area	Ref			Ref			Ref		
High-endemic area	−0.14	−0.46	0.18	−0.07	−0.24	0.10	0.09	−0.38	0.56

## Discussion

This study found that respondents had a medium level of knowledge of COVID-19 and the majority of them had heard of the disease through websites and social media. In comparison, a previous study evaluating parents' knowledge of the H1N1 vaccine found that 66% perceived their knowledge of the H1N1 vaccine to be insufficient at best (16). The difference in the level of knowledge might be due to the increasing amount of information being disseminated online and therefore, its accessibility to young adults in this digital era as evidenced by the main sources of information. Despite having prompt awareness of the disease through the internet, we found that not all of respondents' knowledge of COVID-19 was correct. At the time of this study, the majority of our respondents accurately identified droplets and close contact as the main modes of viral transmission, that we have a test kit to test for the disease, that approved medications to treat the disease are not available, and that a vaccine to prevent the disease is not available. However, some respondents held incorrect beliefs about the disease. The WHO identifies that the virus is mainly transmitted by close contact and respiratory droplets produced through the cough or sneeze of an infected person (6). During the study period laboratory tests exist to screen for the disease, however, no approved pharmacological therapy was available (19). Off-label use of some investigational therapies, such as lopinavir with ritonavir-boost, remdesivir, and herb medicine, have been reported with variable efficacy in the literature (20–24). Additionally, few respondents have also incorrectly identified that a vaccine is available for the disease, although robust research effort in vaccine development is underway (25). Our respondents' knowledge of COVID-19 indicated that although the internet can be an effective way of promoting information, the accuracy of medical information is variable. Only 39% of online medical information is found to be accurate (26). Our findings highlight the importance of increasing education to young adults on the correct modes of transmission and the rapid dissemination of reliable information on the availability of screening and treatment strategies for this disease.

The HBM was used in this study to identify determinants of preventive health behaviors, including washing hands, wearing a face mask, and maintaining social distancing, recommended by China CDC. For wearing a face mask, this study identified cue to action, self-efficacy, and knowledge as significant predictors. Our finding that cue to action was a significant predictor of face mask-wearing was similar to previous studies during the SARS and H1N1 outbreaks (12, 13, 15). Cue to action which significantly predicted face mask-wearing in previous studies included recommendations from local government and family members, being in crowded places and places of vulnerability (e.g., hospitals), situational cues (e.g., seeing people coughing or sneezing, seeing others wearing face masks), and availability of face masks (e.g., availability of face masks provided at no cost) (12, 13, 15). Self-efficacy and knowledge were not always assessed in previous studies. However, when included, they were not significantly correlated with face mask-wearing during the SARS and H1N1 outbreaks (13, 15). This study did not find perceived susceptibility as a significant predictor of face mask-wearing, whereas this was consistently identified as a predictor during the SARS and H1N1 outbreaks (12, 13, 15). This discrepancy could be explained by our respondents' perception that they were at low risk of contracting COVID-19. Since previous studies linking perceived susceptibility to face mask-wearing have found that individuals who feel that they were more vulnerable to the disease were more likely to wear a face mask compared to those who perceived a lower susceptibility to the disease (12, 15). In terms of hand washing, we found cue to action, self-efficacy, and knowledge to be significant predictors, and in terms of social distancing, we found cue to action and self-efficacy to be significant predictors. Previous studies have only assessed preventive health behaviors as a whole and have not studied predictors of individual health behaviors of hand washing and social distancing separately. By identifying predictors of these specific preventive health behaviors, our results could facilitate more targeted educational efforts to enhance the potential adoption of these health behaviors.

Respondents reported that they were moderately or extremely likely to adopt preventive health behaviors. The likelihood to adopt these health behaviors was strongest with face mask-wearing (rated extremely likely), followed by social distancing and hand washing (both rated moderately likely). This might be because respondents perceive face mask-wearing as a social norm and a form of civic responsibility, as illustrated in a previous study conducted in Hong Kong during the SARS outbreak (27). Not wearing face masks during the SARS outbreak reportedly led to stigmatization, social seclusion, and discrimination (27). The adverse social consequences of not wearing a face mask, particularly with its visibility, likely serve as a stronger motivator for its use compared to less visible health behaviors such as hand washing. Based on our identified predictors of these behaviors, improving social distancing and hand washing could be achieved by having local governments and/or public health officials exercise cues to action by recommending young adults adopt these measures through websites or social media. These messages should also aim to increase their confidence in adopting these measures in order to improve self-efficacy. Knowledge also significantly predicted washing hands, but not to practice social distancing. Therefore, to further improve hand hygiene among young adults, public health officials can consider creating online videos and/or infographics illustrating the importance of and the methods to proper hand washing.

Pharmacy students were still staying at home and learning online as all colleges and universities were closed during the study period. This study found that respondents were moderately or extremely likely to adopt preventive health behaviors. The high likelihood of engaging individual preventive measures could support the reopening of colleges and universities in China. Special attention should be paid to washing hands as this is not as visible compared to wearing a face mask. In addition, maintaining social distancing might be challenging in the classroom and cafeteria when college students are back to school. Given the medium level of knowledge of COVID-19 in pharmacy students, continued education through the internet is important. Local and school authorities should make clear guidance to support individuals to adopt preventive health behaviors to reduce the risk of community transmission of COVID-19 in colleges and universities.

This study has some limitations worth mentioning. First, this study only included pharmacy students, therefore the results cannot be generalizable to the general public. Second, due to the use of a questionnaire for data collection, the actual level of knowledge and preventive health behaviors might be different from what has been reported. Third, because only components of the HBM were evaluated, there might be other factors that could motivate health behaviors that were not accounted for in this study. However, the HBM is one of the most widely used belief-based psychosocial theories and has been extensively used to assess preventive health behaviors in previous virus outbreaks.

## Conclusions

This study found that future pharmacists in high and low-endemic areas in China had a medium level of knowledge of COVID-19. Further efforts to improve their knowledge can be focused on providing timely, reliable, and accurate information on the internet. In the HBM, cue to action, self-efficacy, and knowledge were significant predictors of preventive health behaviors of washing hands and wearing a face mask, whereas only cue to action and self-efficacy were significant predictors of maintaining social distancing. To potentially improve the adoption of face mask-wearing, hand washing, and social distancing as appropriate individual preventive measures, targeted educational efforts should be implemented to enhance cue to action, self-efficacy, and knowledge. To prepare for reopening schools, continued monitoring of individuals' knowledge and behavioral responses to COVID-19 is warranted.

## Data Availability Statement

The raw data supporting the conclusions of this article will be made available by the authors, without undue reservation.

## Ethics Statement

The studies involving human participants were reviewed and approved by University of Tennessee Health Science Center. The ethics committee waived the requirement of written informed consent for participation.

## Author Contributions

GL and JY: conceptualization, methodology, and writing, SH: methodology and writing, RS: methodology and analysis, ML: conceptualization, methodology, analysis, writing, and supervision. All authors contributed to the article and approved the submitted version.

## Conflict of Interest

The authors declare that the research was conducted in the absence of any commercial or financial relationships that could be construed as a potential conflict of interest.
